# Temperature-controlled laminar airflow in severe asthma for exacerbation reduction (The LASER Trial): study protocol for a randomised controlled trial

**DOI:** 10.1186/s13063-015-1134-y

**Published:** 2016-01-08

**Authors:** Will Storrar, Carole Fogg, Tom Brown, Paddy Dennison, Ly-Mee Yu, Ann Dewey, Ramon Luengo-Fernandez, Tara Dean, Najib Rahman, Adel Mansur, Peter H. Howarth, Peter Bradding, Anoop J. Chauhan

**Affiliations:** Portsmouth Hospitals NHS Trust, Portsmouth, UK; University Hospital Southampton NHS Foundation Trust, Southampton, UK; Centre for Statistics in Medicine, Oxford University, Oxford, UK; University of Portsmouth, Portsmouth, UK; Health Economics Research Unit, Nuffield Department of Population Health, University of Oxford, Oxford, UK; Oxford Respiratory Trials Unit, University of Oxford, Oxford, UK; Heart of England NHS Foundation Trust, Birmingham, UK; University Hospitals of Leicester NHS Trust, Leicester, UK

**Keywords:** Severe asthma, Temperature-controlled laminar airflow, Exacerbation, Quality of life, Cost effectiveness

## Abstract

**Background:**

Asthma affects more than 5 million patients in the United Kingdom. Nearly 500,000 of these patients have severe asthma with severe symptoms and frequent exacerbations that are inadequately controlled with available treatments. The burden of severe asthma on the NHS is enormous, accounting for 80 % of the total asthma cost (£1 billion), with frequent exacerbations and expensive medications generating much of this cost.

Of those patients with severe asthma, 70 % are sensitised to indoor aeroallergens, and the level of exposure to allergens determines the symptoms; patients exposed to high levels are therefore most at risk of exacerbations and hospital admissions.

The LASER trial aims to assess whether a new treatment, temperature controlled laminar airflow (TLA) delivered by the Airsonett™ device, can reduce the frequency of exacerbations in patients with severe allergic asthma by reducing exposure to aeroallergens overnight.

**Methods:**

This multicentre study is a placebo-controlled, blinded, randomised controlled, parallel group trial. A total of 222 patients with a new or current diagnosis of severe allergic asthma will be assigned with a random element in a 1:1 ratio to receive either an active device for one year or a placebo device. The primary outcome is the frequency of severe asthma exacerbations occurring over a 12-month period, defined in accordance with the American Thoracic Society/European Respiratory Society (ATS/ERS) guidelines. Secondary outcomes include changes in asthma control, lung function, asthma-specific and global quality of life for participants and their carers, adherence to intervention, healthcare resource use and costs, and cost-effectiveness. Qualitative interviews will be conducted to elicit participant’s and their partner’s perceptions of the treatment.

**Discussion:**

Effective measures of allergen avoidance have, to date, proved elusive. The LASER trial aims to address this. The study will ascertain whether home-based nocturnal TLA usage over a 12-month period can reduce the frequency of exacerbations and improve asthma control and quality of life as compared to placebo, whilst being cost-effective and acceptable to adults with poorly controlled, severe allergic asthma. The results of this study will be widely applicable to the many patients with allergic asthma both in the UK and internationally.

**Trial registration:**

Current controlled trials ISRCTN46346208 (Date assigned 22 January 2014).

## Background

Asthma affects over 5.4 million people in the United Kingdom, with nearly 500,000 experiencing severe symptoms and frequent exacerbations that are inadequately controlled with available treatments [1, 2]. The burden of severe asthma on the National Health Service (NHS) is enormous, accounting for 80 % of the total asthma cost (£1 billion; https://www.asthma.org.uk/about/media/facts-and-statistics/) with frequent exacerbations and expensive medications generating much of this cost [3].

Current treatments including oral corticosteroids, ‘steroid-sparing’ immuno suppressants and monoclonal antibody therapies are often of limited efficacy and have potentially serious side effects (steroids, immunosuppressive agents) or are prohibitively expensive (monoclonal antibodies). The adverse effects of long-term oral steroids include adrenal suppression, decreased bone mineral density, diabetes, and increased cardiovascular mortality [4]. The anti-IgE treatment Omalizumab™ has been shown to reduce exacerbations by up to 50 % [5] and improve the quality of life in severe allergic asthma but can cost as much as £26,640 per year, [6], which is substantially more than the current annual rental cost of a TLA device (£2,088).

The 2011 British Thoracic Society (BTS) and Scottish Intercollegiate Guidelines Network (SIGN) national asthma guidelines [7] and 2010 WHO consultation on severe asthma [8] have highlighted an urgent need for research in severe asthma, acknowledging the limitations of available treatments in severe asthma and the dearth of clinical trials upon which to base management recommendations.

More than 70 % of severe asthmatic patients are sensitised to common aeroallergens and/or moulds [9], and the level of allergen exposure determines symptoms; those exposed to high allergen levels are at an increased risk of exacerbations and hospital admissions [10–13]. Allergen avoidance has been widely recognised as a logical way of treating these patients [14]. In controlled conditions, long-term allergen avoidance in sensitised asthmatics reduces airway inflammation with consequent symptomatic improvement, further supported by high-altitude, clean-air studies [15–17].Unfortunately, effective methods of allergen reduction have proved elusive [18, 19] with current measures being unable to reduce allergen load sufficiently to yield a consistent clinical improvement, thus leaving a significant gap in the potential strategies for reducing asthma severity through allergen reduction.

At night, airborne particles are carried by a persistent convection current established by the warm body, transporting allergens from the bedding area to the breathing zone [20]. Proof-of-concept studies have shown the TLA device reduces the total number of airborne particles and significantly reduces the increase in particles generated when turning in bed at night [21]. When compared to a best-in-class traditional air cleaner, TLA is able to reduce exposure to potential allergens by a further 99 % [22]. We postulate that this highly significant reduction in nocturnal exposure, targeted to the breathing zone, explains why TLA may succeed in an area where so many other measures, including air filters, have failed.

The TLA device, when compared to a placebo, has proven efficacy on asthma-related quality of life and bronchial inflammation (measured by exhaled nitric oxide) in a pan-European multicentre Phase III study, [23] (n = 282, age range 7 to 70 years). The greatest benefit was seen in the more severe asthma patients who required higher intensity treatment and in patients with poorly controlled asthma. Whilst not powered to ascertain an effect on exacerbations, a post-hoc analysis showed a decreased exacerbation rate in more severe patients treated with TLA compared with placebo, with a trend towards significance (mean 0.23 TLA; 0.57 placebo *P* = 0.07).

A further pragmatic, patient-centred RCT of this novel non-pharmacological treatment in severe allergic asthma is now warranted.

## Methods

### Study objectives

#### Primary aim

The primary aim of the trial is to determine whether home-based nocturnal treatment with a temperature-controlled laminar airflow (TLA) device can reduce the frequency of severe asthma exacerbations over a 1-year period. Exacerbations are defined according to the American Thoracic Society (ATS) and European Respiratory Society (ERS) criteria, that is, an acute deterioration in asthma that requires treatment with systemic corticosteroids.

#### Secondary aims

The secondary aims include assessing the impact of nocturnal TLA treatment on additional aspects of asthma control including symptoms, reliever use, airway obstruction, and on the risk of future adverse asthma outcomes, including an accelerated decline in lung function and the side-effects of treatment over the 12-month period. The impact of the treatment on the quality of life of the patients and their carers will be assessed using the Asthma Quality of Life Questionnaire (AQLQ) and a generic health-related quality of life questionnaire (EQ-5D-5L) for trial participants and the Adult Carer Quality of Life questionnaire (ACQoL) for participant’s adult carers. The impact of the treatment on both the NHS and social costs, that is, healthcare utilisation, will be measured using patient questionnaires and a review of patient’s records and education/work days lost, which will be assessed using the Work Productivity Activity Impairment questionnaire (WPAI) in both participants and their adult carers. These data will be further analysed to assess the cost-effectiveness of the intervention, using a cost-utility analysis to determine the incremental cost per quality-adjusted life year (QALY) gained. Finally, we aim to qualitatively evaluate the perceptions, values and opinions of the participants and their partners relating to the device to identify potential modifications that may improve patient acceptance and will inform future implementation of the device within the NHS.

#### Exploratory aims

We will explore the possible associations of patient and environmental factors that may be associated with a treatment response.

### Study design and setting

**Fig. 1:**
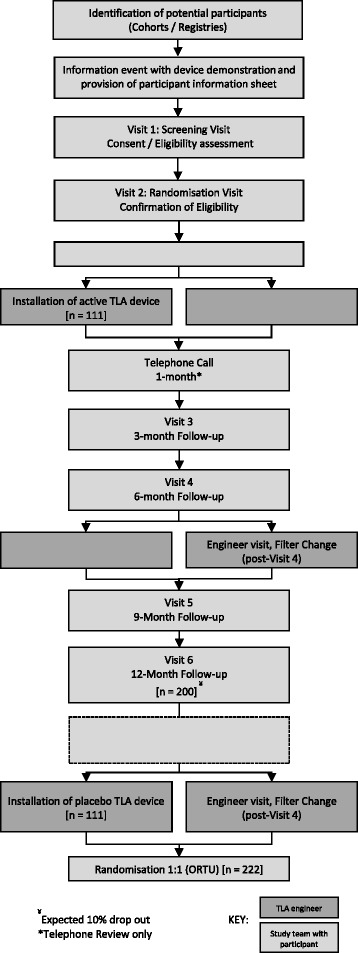
Study flow diagram

This is a multi-centre randomised, double-blind, placebo-controlled parallel group trial comparing an active TLA device with a placebo device over a 12-month period. The device is installed in the participant’s home and each participant is required to attend six study visits at their usual referral hospital. Qualitative and health economics methods have been incorporated to the trial. A summary of the study design is illustrated in Fig. 1. The trial includes a 4-month internal pilot to assess the recruitment and retention of participants, data collection methods and quality, and participant and partner experience of the trial through qualitative methods. Study recruitment commenced in May 2014. The plan is to conduct the trial in as many as 15 centres in the UK.

### Selection of participants

#### Recruitment and informed consent

As both new and prevalent severe asthma patients can be included in the trial, a variety of approaches will be used for recruitment. Some referral centres have cohorts of well-characterised asthma patients with detailed clinical and biological information, who can be contacted directly by the research team and invited to local study information events or a screening visit in the clinic. Potential participants will also be identified through existing clinic registers and referrals to severe asthma clinics. Following advertisement and launch of the website (http://www.lasertrial.co.uk/), a mechanism will be in place for patients to self-refer to the study team for screening. Participant’s adult carers and or their adult partners will also be requested to attend events/visits where possible because they will be recruited to participate in the trial. Participant information sheets will be made available to potential participants prior to their screening visit. Separate information sheets for Carers and Partners will also be available. Participants invited to the screening clinics will have been ‘pre-screened’ for known exclusion criteria such as age, smoking, co-morbidities and concomitant use of excluded asthma treatments. Informed consent for screening and trial procedures will be obtained from participants at Screening Visit 1. Informed consent from partners and carers will be obtained at either Screening Visit 1 or Randomisation Visit 2.

Procedures performed at Screening Visit 1 include the following:

1. Baseline pre-bronchodilator spirometry and bronchodilator reversibility testing.
2. Skin prick testing (SPT) to a panel of indoor aeroallergens (dog, cat, house dust mite, and fungi).
3. Blood tests (peripheral blood eosinophil count and total serum IgE in all cases and specific serum IgE testing if the SPT is not available or cannot be performed).
4. Asthma Control Questionnaire (ACQ) completion.

Participants will be provided with an electronic PEF meter to measure morning and evening PEF (prior to taking asthma medications) for 2 weeks prior to Randomisation Visit 2. Participants will also complete an asthma control diary for 2 weeks prior to Randomisation Visit 2.

Participants will be asked to return with this information after 2 weeks, so their adherence and ability to perform daily readings can be assessed along with the stability of their asthma prior to randomisation. Eligibility criteria will be confirmed at Randomisation Visit 2. Patients who experience an exacerbation within the 2-week run-in period may be re-screened after a further 2 weeks if they wish.

### Inclusion criteria

To be included, participants must meet the following criteria:

1. Adult (aged 16–75 years inclusive)
2. A clinical diagnosis of asthma for ≥ 6 months supported by evidence of *any one* of the following:
  - Airflow variability with a mean diurnal peak expiratory flow (PEF) variability >15 % during the baseline 2-week period or a variability in FEV_1_ of > 20 % across clinic visits within the preceding 12 months, with concomitant evidence of airflow obstruction (FEV_1_/FVC ratio < 70 %).
  - Airway reversibility with an improvement in FEV_1_ by ≥ 12 % or 200 ml after inhalation of 400 μg of salbutamol via a metered dose inhaler and spacer at first study visit or within the preceding 12 months.
  - Airway hyper-responsiveness demonstrated by methacholine challenge testing with a provocative concentration of methacholine required to cause a 20 % reduction in FEV1 (PC_20_) of ≤8 mg/ml or equivalent test.
3. Severe asthma (ATS/ERS guideline, 2013 [24]) defined as follows:
  - Requirement for high-dose inhaled corticosteroids (ICS) (≥1000 μg/day beclomethasone (BDP) or equivalent) plus a second controller (long-acting ß2-agonist or anti-muscarinic, theophylline, or leukotriene antagonist), and/or systemic corticosteroids.
  - If on maintenance corticosteroids, the maintenance dose must have been stable for 3 months; this excludes any interim need for short-term steroid bursts to treat exacerbations.
4. Poorly controlled asthma demonstrated by BOTH of the following:
  - Two or more severe asthma exacerbations, requiring systemic corticosteroids ≥ 30 mg prednisolone or equivalent daily (or ≥ 50 % increase in dose if maintenance 30 mg prednisolone or above), for 3 or more days, during the previous 12 months, despite the use of high-dose inhaled corticosteroids (ICS) and additional controller medication.
  - ACQ (7-point) score > 1 at Screening Visit 1 and Randomisation Visit 2.
5. Atopic status, defined as sensitisation to ≥ 1 perennial indoor aeroallergen (including house dust mite, domestic pet or fungi) to which they are likely to be exposed during the study, demonstrated by a positive skin prick test (wheal diameter ≥ 3 mm more than negative control) or specific IgE ≥ 0.35 IU/L).
6. Exacerbation-free and taking stable maintenance asthma medications (not including short-acting bronchodilator or other reliever therapies) for at least 2 weeks prior to Screening Visit 1.
7. Exacerbation free and taking stable maintenance asthma medications (not including short-acting bronchodilator or other reliever therapies) in the period between Screening Visit 1 and Randomisation Visit 2.
8. Able to use the TLA device during sleep on at least 5 nights per week (excluding holidays).
9. Able to give written informed consent prior to participation in the trial and able to comply with the trial requirements.

### Exclusion criteria

1. Current smokers or ex-smokers abstinent for < 6 months.
2. Ex-smokers with ≥ 15 pack/year smoking history.
3. Partner who is a current smoker and smokes within the bedroom where the TLA device is installed.
4. TLA device cannot be safely installed within the bedroom.
5. Intending to move out of study area to an area not within reach of a participating referral hospital within the follow-up period.
6. Documented poor treatment adherence.
7. Occupational asthma with continued exposure to known sensitising agents in the workplace.
8. Previous bronchial thermoplasty within 12 months of randomisation.
9. Treatment with Omalizumab (anti-IgE) within 120 days of randomisation.
10. Using long-term oxygen, continuous positive airway pressure (CPAP) or non-invasive ventilation (NIV) routinely overnight.
11. Uncontrolled symptomatic gastro-oesophageal reflux that may act as a persistent asthma trigger.
12. Presence of clinically significant lung disease other than asthma, including smoking-related chronic obstructive pulmonary disease (COPD), bronchiectasis associated with recurrent bacterial infection, allergic broncho-pulmonary aspergillosis (mycosis), pulmonary fibrosis, sleep apnoea, pulmonary hypertension, or lung cancer, that in the opinion of the Principal Investigator is likely to be contributing significantly to the participant’s symptoms.
13. Clinically significant co-morbidity (including cardiovascular, endocrine, metabolic, gastro-intestinal, hepatic, neurological, renal, haematological and malignant conditions) that remains uncontrolled with standard treatment.
14. Currently taking part in other interventional respiratory clinical trials.

### Randomisation and blinding

Participants will be assigned with a random element in a 1:1 ratio between the intervention and control groups using a centralised randomisation system, Sealed Envelope™. A nondeterministic minimisation algorithm will be used to facilitate balanced allocation of participants across the two treatment groups for 1) clinical site, 2) prevalent vs. incident case and the following prognostic factors at baseline: 3) exacerbation frequency in the previous 12 months (2, 3, or ≥3), 4) use of oral corticosteroids (yes/no) and 5) pre-bronchodilator FEV1 (>50 % predicted yes/no).

Sealed Envelope™ will have been provided with a list of TLA product serial numbers by the manufacturing team based in Sweden and will allocate a specific TLA product to the participant. A secure e-mail notification will be sent immediately to the local trial team to confirm randomisation. A secure e-mail will be sent to the UK-based engineering team. This will include the participant’s trial number, TLA product serial number, and an exclusive, password protected, link for the engineering team to log in and access the participant’s contact details. The engineering team will then contact the participant (within 72 hours of Randomisation Visit 2) to arrange device delivery and installation without disclosing the treatment allocation to the participant. All study team members and the participants will be blind to which device they have received.

### Study intervention

**Fig. 2:**
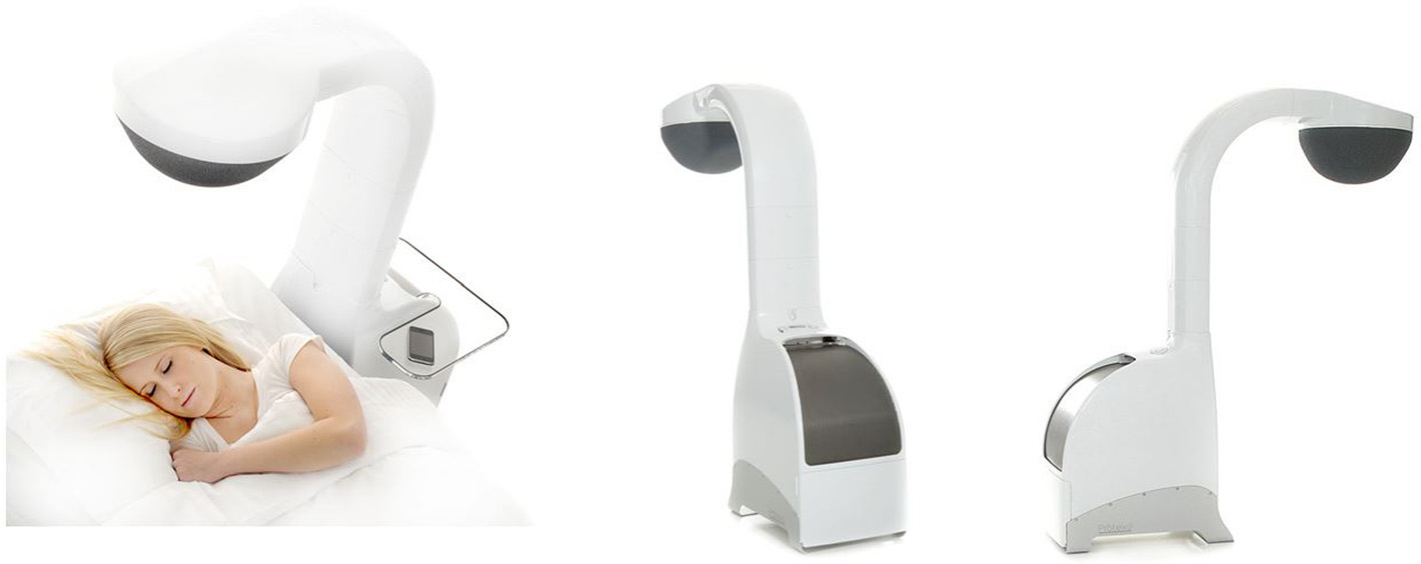
The temperature-controlled Laminar Airflow (TLA) device (Airsonett.)

The active TLA device (Airsonett™) significantly reduces nocturnal allergen exposure by filtering ambient air through a high efficiency particulate air filter, slightly cooling (0.5-0.8° C) and ‘showering’ it over the participant during sleep (Fig. 2). The reduced temperature allows the filtered air to descend in a laminar stream, displacing allergen-rich air from the breathing zone reducing allergen exposure without creating draft or dehydration. The device is installed next to the participant’s bed by a qualified engineer from the company. The device is pre-programmed to turn on and off at times specified by the trial participant but can be turned on and off by manual override if the participant wishes to use the device for an extended period or turn the device off when not in use. The device is easy to use with no identified safety concerns in previous trials. The device is CE marked and licensed for use in the UK for allergic asthma. The device uses the same amount of electricity as a 60 W light bulb and has an anticipated life-span of 5 years with filter changes required every 6 months. Participants will be asked to sleep under the device for at least 5 nights per week except when on holiday during the 12-month treatment period. Participants will record whether they have used the device or not and number of hours used on a daily basis, recorded in a ‘LASER’ diary. Any additional hours used during the day will also be recorded in the diary.

### Control arm

The control treatment consists of a placebo device that appears physically identical to the intervention device to the study participant. These devices are adjusted to deliver isothermal air, instead of slightly cooled air, and holes in the filter effectively bypass it whilst still maintaining an equivalent sound and airflow level to an active device. This allows the placebo device to deliver a laminar flow of non-filtered, non-descending, isothermal air which, when mixed with the warm body convection, will ascend towards the ceiling and thus have no effect on the normal air flow pattern around the breathing zone. No difference exists in the air delivery rate, perceived air movements or sound level between an active or placebo device. The human body is not able to detect an absolute temperature difference of 0.75 °C, and as such, no perceptible temperature difference exists when sleeping beneath an active or a placebo device. Electricity usage is the same as for active devices, and the filter is changed at 6 month intervals.

### Measurement of primary outcome

The primary outcome, severe asthma exacerbations, are defined according to ATS/ERS guidelines [25] as a worsening of asthma requiring systemic corticosteroids ≥ 30 mg prednisolone or equivalent daily (or ≥ 50 % increase in dose if maintenance 30 mg prednisolone or above), for 3 or more days. Courses of corticosteroids separated by ≥ 7 days will be treated as separate severe exacerbations. Based on this, an ‘exacerbation-dose’ of systemic corticosteroids is defined as ≥ 30 mg prednisolone or equivalent daily if not on a maintenance systemic corticosteroid treatment or ≥ 50 % increase in dose if maintenance dose is 30 mg prednisolone or above.

Participants will be asked to start an exacerbation diary when starting an ‘exacerbation-dose’ of systemic corticosteroids (participant specific ‘exacerbation-dose’ is defined at Randomisation Visit 2). The exacerbation diary will include PEF measurements (using the trial electronic PEF device), oral corticosteroid dose, reliever medication use, and nocturnal asthma symptoms. Participants will be asked to report severe exacerbations to their local site trial team as soon as possible after onset via a dedicated telephone line or a secure NHS e-mail account. Wherever possible, participants will be asked to attend an exacerbation review with their local trial team within 72 hours to corroborate the exacerbation, at which the local trial team will complete the case report form (CRF) using the exacerbation diary. The definition of a severe asthma exacerbation with *any one* of the following additional criteria must be met:

1. An associated decrease in morning PEF compared to maximum morning PEF achieved at baseline.
2. A 50 % increase in reliever medication on at least 2 of 3 successive days compared to baseline.
3. Increased nocturnal wakening.

If participants are not able to attend for an exacerbation review, the exacerbation diary will be collected at the next follow-up visit.

### Measurement of secondary outcomes

Outcomes reflecting current asthma control will be measured in the clinic at 3, 6, 9 and 12 months, and will include (i) measures of lung function (pre-bronchodilator FEV_1_, mean morning pre-bronchodilator peak expiratory flow (PEF) rate over 2 weeks prior to the visit, fractional concentration of exhaled nitric oxide (F_E_NO)); (ii) measures of the risk of future adverse asthma outcomes (severe exacerbations, systemic corticosteroid use over the 12 months, post-bronchodilator FEV_1_ at 12 months); (iii) symptom control patient-reported scores Asthma Control Questionnaire (ACQ), Asthma Control Diary, and Sino-Nasal Outcome Test (SNOT-22) score; and (iv) health-related quality of life for both the study participant (standardised asthma quality of life questionnaire (AQLQ(S)) and EuroQol 5-dimension 5-level questionnaire (EQ5D-5L) and the carer (Adult Carers Quality-of-Life questionnaire (AC-QoL) at 12 months only). Responses to the EQ-5D will be converted into utilities using UK population tariffs [26] and combined with survival information to generate quality-adjusted life years (QALYs). A global evaluation of treatment effect (GETE score) will be recorded at 12 months.

The usage of the device will be measured from participant reports at 3, 6, 9 and 12 months and also by an engineer at the time of device filter change at 6 and 12 months.

We will assess the costs associated with the TLA device (including acquisition, installation and servicing). In addition, using patient questionnaires and review of patients’ records, health and social care resource use data will be collected. Resource use data will be valued using NHS and social care costs schedules [27]. Work productivity and activity impairment (WPAI) questionnaires will be given to the participant (WPAI - Asthma) at 3, 6, 9 and 12 months, and to the carer (WPAI – Care Giver) if applicable at 12 months.

Thematic analysis of qualitative data will elicit information on the participant and their partner’s perception of the treatment device.

### Exploratory analysis

Device adherence, objective markers of bronchial and systemic allergy and inflammation, lung function measures, asthma and rhinitis control, quality of life and indoor air quality assessments will be analysed to determine factors that may be associated with treatment response.

### Study visit schedule

The study visit schedule consists of two visits during the screening period to assess eligibility and four visits during the 12-month treatment period at 3-month intervals, 3, 6, 9 and 12 months post-randomisation (Table 1).

**Table 1:**
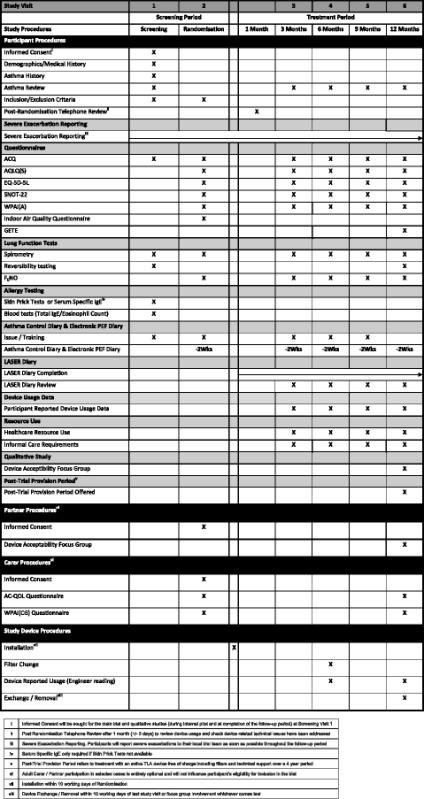
Study visit schedule

^i^Informed Consent will be sought for the main trial and qualitative studies (during internal pilot and at completion of the follow-up period) at Screening Visit 1

^ii^Post Randomisation Telephone Review after 1 month (+/- 3 days) to review device usage and check device related technical issues have been addressed

^iii^Severe Exacerbation Reporting. Participants will report severe exacerbations to their local trial team as soon as possible throughout the follow-up period

^iv^Serum Specific IgE only required if Skin Prick Tests not available

^v^Post-Trial Provision Period refers to treatment with an active TLA device free of charge including filters and technical support over a 4 year period

^vi^Adult Carer / Partner participation in selected cases is entirely optional and will not influence participant’s eligibility for inclusion in the trial

^vii^Installation within 10 working days of Randomisation

^viii^Device Exchange / Removal within 10 working days of last study visit or focus group involvement whichever comes last

At the first visit (Screening Visit 1), after consent, the following information is collected: participant’s demographics, past medical history, occupational history, participant’s asthma history including exacerbation history and a review of current asthma symptoms and current medications, bronchodilator reversibility testing and allergy testing (skin prick tests or serum specific IgE) are performed, Blood samples are taken for total IgE and peripheral blood eosinophil count. Participants are asked to complete an ACQ and are issued with an electronic peak expiratory flow meter for use during the trial.

At the second visit (Randomisation Visit 2), the asthma control diary and peak flow diary is collected. Participants complete the following questionnaires: ACQ, AQLQ, EQ-5D-5L, WPAI-A, SNOT-22 and the LASER Indoor Air Quality Questionnaire. Pre-bronchodilator spirometry is performed, and FENO is measured. Allergy testing is performed if this was not possible at Screening Visit 1. Participants are issued with the following: (i) a LASER diary for daily recording of overnight device use (hours,) additional device use (hours,) time off work as a result of asthma (hours) and dose of oral corticosteroids (mg); (ii) exacerbation diary cards for completion at the onset of an exacerbation throughout the trial (including PEF, steroid dose, reliever medication use and nocturnal wakening); and (iii) an Asthma Control Diary for completion prior to their next follow-up visit (including morning pre-bronchodilator PEF.) Participants will also be issued a Resource Use Log and a participant identification card.

Participants whose partner and/or carer wish to participate in the trial are asked to attend together for either visit 1 or visit 2 so that Carer/Partner consent can be obtained. Partners are asked to consent for participation in the qualitative study and carers are asked to consent to completion of questionnaires at visit 2 and at the 12-month follow-up visit (ACQoL and WPAI-CG).

One month following the Randomisation Visit 2, participants are contacted by telephone for their 1-month telephone review, where device delivery and installation will be confirmed, and any problems with the device or trial can be addressed.

Follow-up visits are held at 3-, 6-, 9- and 12-months post-randomisation. Participants collect 2 weeks of peak flow recordings and an asthma control diary prior to each follow-up visit. At the follow-up visits, participants complete questionnaires (ACQ, AQLQ, EQ-5D-5L, SNOT-22 and WPAI-A) and perform pre-bronchodilator spirometry and FENO. At each follow-up visit, exacerbation history is reviewed along with self-reported device usage using the LASER diary and healthcare resource use log. Device reported use is also documented at each follow-up visit.

At the 12-month follow-up visit, a GETE questionnaire will also be completed.

Participants will report severe exacerbations to their local trial team throughout the trial, and if possible, face-to-face reviews will be arranged to corroborate the diagnosis of a severe exacerbation. If a face-to-face review is not possible, then a telephone review will be recorded.

### Safety reporting

Participants will be asked about the occurrence of any adverse events (AEs) (using the definition given in the International Conference on Harmonisation - Good Clinical Practice (ICH GCP) [28]) at each follow-up visit and will be asked to report AEs to their local trial team between visits. Only AEs that have a reasonable possibility of being attributable to the device (that is an Adverse Device Effect (ADE)) and any other AE considered to be of clinical significance by the local principal investigator as causing harm to the patient will be recorded. ADEs also include any event resulting from insufficiencies or inadequacies in the instruction for use or deployment of the device and includes any event that is a result of a user error.

Events exempt from recording are those related to worsening asthma control or the main study outcomes, namely the following:

1. An increase in rescue medication usage.
2. Additional courses of steroids for asthma exacerbations.
3. Increased unscheduled healthcare usage including GP and Emergency Department visits for deteriorations in asthma control.
4. Time off work, College or University due to worsening asthma control.
5. Hospitalisation due to asthma exacerbation.
6. Increased number or intensity of asthma exacerbations.

All serious adverse events (SAEs) and serious adverse device effects (SADEs), defined as any untoward medical occurrence seen in a patient that can be attributed wholly or partly to the device and resulted in any of the characteristics or lead to the characteristics of a Serious Adverse Event) will be recorded on a serious adverse event form and be reported within 24 hours to the Oxford Respiratory Trials Unit (ORTU). A second medical assessment of all reported SAE/SADEs will then be performed, and if considered by either the PI or ORTU to be possibly, probably or definitely related to the device (SADE), will be expedited to the Sponsor, REC and device manufacturer within 7 days of ORTU becoming aware of the event, if fatal or life threatening, or otherwise within 15 days. Listings of adverse events will be provided to the Data Safety Monitoring Committee (DSMC) and the Sponsor when requested. The DSMC will report to the TSC and Sponsor regarding the safety profile of the trial.

### Statistics

The study will be reported in accordance with the Consolidated Standards of Reporting Trials statement and ICH Guidelines for Good Clinical Practice. All study data will be managed by ORTU using a bespoke database created using OpenClinica Enterprise Edition software (OpenClinica LLC, Waltham MA, USA). Confidentiality of participant data will be assured according to GCP.

### Sample size

Based on an estimated rate of two severe asthma exacerbations per participant over the 12-month period in the placebo group, a minimum of 222 participants (111 per group) will be required to provide 80 % power (at 5 % two-sided significance level) to detect a clinically meaningful 25 % reduction in the average exacerbation rate in the group using the TLA device.

This sample size is based on a Poisson regression model, with the treatment group as the covariate and a 10 % overall dropout rate. A review of comparative interventions of proven efficacy in severe asthma gave effect sizes ranging from 21 to 63 %, with a mean of 41 %. Given that this is a pragmatic trial where we expect our intervention to be less effective than an efficacy trial, we have chosen a deliberately more conservative effect size of 25 %. This represents on average, one less severe exacerbation every 2 years.

### Clinical outcomes analysis

The primary statistical analysis will be carried out on the basis of intention-to-treat (ITT). The primary efficacy endpoint in this study, the rate of clinically significant exacerbations over the 12-month period, will be analysed using a Poisson regression model to compare the rate of asthma exacerbations between the two groups with log of time used as an offset variable. Further analysis will adjust for the baseline characteristics including the ACQ score, age, body mass index (BMI) and sex.

Continuous secondary endpoints which involve repeated measures at baseline, 3, 6, 9, and 12 months (including measures of lung function, composite asthma control scores and health-related quality-of-life measures) will be analysed using longitudinal analysis methods, including mixed effect models to determine any effect of the TLA device over time. Continuous variables with only baseline and 12 months data, (including lung function and carer quality of life measures) will be analysed by analysis of covariance (ANCOVA) of the 12-month outcome adjusted where appropriate for baseline, minimisation factors and other important factors as detailed for the primary outcome.

Kaplan–Meier curves and log-rank test will be used to compare the time to first asthma exacerbation between the two groups. Cox proportional hazards models will be used to evaluate the effect of TLA device on the time to first asthma exacerbation, adjusting for the same covariates as in the primary analysis. Since the analysis of only time to first exacerbation leaves out much of the data, analysis incorporating multiple time-to-event (recurrent exacerbations) methods will also be used, including the Andersen–Gill extension.

The proportion of participants experiencing severe exacerbations over the 12-month follow-up period will be compared using a continuity-corrected Chi-squared test. The duration of severe exacerbations, the total number of days in an exacerbation state over the 12-month follow-up period, and the number of health care utilisations will be compared between the two groups using two-sample independent t-tests.

Exploratory sub-group analyses will include an assessment of factors associated with an improved treatment response including objective markers of bronchial and systemic allergy and inflammation, lung function, asthma and rhinitis control, quality of life and level of indoor air quality. The predictive effect of the biomarkers on exacerbations will be assessed by including the biomarker as an independent covariate together with the biomarker-treatment interaction using Poisson regression modelling in a multivariate framework (as described for the primary outcome).

### Missing data

We will attempt to minimize the missing data due to item non-response during the long follow-up period by ensuring timely contact with the participants and robust follow-up procedures. The expected participant’s dropout has already been factored into the sample size calculation. Missing data will be reported with reasons given where available, and the missing data pattern, explored. In order to be consistent with the ITT, missing data for the primary endpoint will be imputed using multiple imputation (MI) techniques. Our imputation model will be sufficiently general to include all the baseline variables thought to be important predictors of the response indicator of each target variable to be imputed. This will improve the validity of the imputation model under the missing at random (MAR) assumption on which the MI is based. In addition, an ignorable likelihood-based analysis will be applied for the mixed effect models.

### Interim analysis and criteria for early study termination

No interim analysis is planned; however, the DSMC will perform regular reviews of all study outcome and adverse event data to ensure that there is no difference in rates of hospitalisation or exacerbation in either group. The DSMC will determine final criteria for early study termination, which may be based on clear-cut evidence of worsened safety in one of the trial arms, and in the case of evidence beyond reasonable doubt of clear-cut benefit in the primary outcome measure, an effect size which would change clinical practice in the presence of the current literature and understanding of the disease area.

### Economic evaluation

The perspective adopted in the economic evaluation will be that of the National Health Service (NHS) and social services; therefore, productivity losses and over-the-counter medication costs will not be included in the base case analysis. However, in a sensitivity analysis, we will assess the impact of including these costs on the cost-effectiveness results.

A within-trial cost-utility analysis will explore the incremental cost per QALY gained by TLA usage when compared to sham-TLA usage. Cost and effect results will be reported as means with standard deviations, with mean differences between the two patient groups reported alongside 95 % confidence intervals (CI). Incremental cost-effectiveness will be calculated by dividing the difference in costs by the difference in effects. Uncertainty around the incremental cost-effectiveness ratio (ICER) will be explored using non-parametric bootstrapping [29].

Building on the results of the trial and subsequent cost-effectiveness analysis, a Markov model will be constructed to determine the costs and outcomes, over the life-time of the patient, of TLA usage. The model structure will be informed by results from this trial, and from previously published studies, with experts within the team advising on the final structure of the model. The analysis will determine the cost per life year gained and cost per QALY gained when nocturnal TLA treatment is compared to placebo. The model will be run over the patient lifetime, with costs and benefits discounted at a rate of 3.5 %. Probabilistic sensitivity analyses will be conducted to deal with uncertainty in model parameters and cost-acceptability curves presented, [30] and will be extended to consider the application of value of information (VoI) techniques, which are included in economic evaluations to inform policy decisions about the value of further research.

### Qualitative analysis

Informed consent for participation in the qualitative study will be sought at Screening Visit 1. All participants taking part in the LASER trial will be invited to take part in the qualitative study, although this is not mandatory.

During the initial 4-month pilot phase of the trial, telephone interviews will be conducted with participants. Telephone interviews will follow a topic guide developed in conjunction with the PPI members and will focus on the study procedures to elicit aspects of the study that may be improved. We will also gather information about participant’s experience of using the TLA device. The qualitative interviews will help to identify potential barriers to recruitment, treatment adherence and device acceptability. Telephone interviews will be digitally recorded and transcribed verbatim for analysis. Results of the telephone interviews will be used to inform subsequent development and refinement of trial processes. The interviews will also be used to strengthen the frequently asked questions area of the website. Key themes identified during the telephone interviews will be used to inform the topic guide for the later focus group interviews.

Following completion of their 12-month treatment period, participants who have consented to participation in the focus group interviews will be invited to attend. Focus groups will be held for both satisfied and non-satisfied users. All interviews will be digitally recorded, transcribed verbatim and entered into NVivo10, a qualitative software package for systematic and transparent data management. Contributions by participants will remain anonymous. An identification using a pseudonym will be assigned to each participant at recruitment. No ‘real’ names will be included in any reports. Care will be taken to always ensure any direct quotes used in study report or papers to illustrate the findings will not be directly attributable to individuals.

We will use Framework Analysis, a three-stage analytic process, to analyse the data. This involves identifying initial themes by indexing the content of the data; this then guides the formation of a framework within which transcribed material is synthesised. Key categories are then identified to help describe the data. Finally, patterns of association are explored and attempts made to explain why those patterns occur.

### Patient public involvement

Patient public involvement has been sought throughout. PPI members have been engaged from the initial grant application to trial design and delivery and continue to play an active role in areas such as development of the trial website. PPI members will continue to be involved as the trial progresses, helping to guide the focus group interview schedule and in the dissemination of the results.

### Ethics

A favourable ethical approval for this study was granted by the National Research Ethics Service (NRES) Committee, South Central – Berkshire (reference 14/SC/0092) on 26 February 2014. The trial will be conducted according to the Declaration of Helsinki [31].

The main ethical issue in this trial is the provision of a placebo device for a 1-year period. The trial team considered alternative ‘add-on’ treatments in severe asthma (for example, omalizumab and bronchial thermoplasty), but these vary greatly in indication, use and delivery; are not suitable for every patient; and would therefore not be able to be used consistently or safely in an ‘active’ control group. As the TLA device is unique in its design and purpose and is intended as an addition to standard asthma treatment, the use of a placebo device is warranted here and was deemed acceptable by the service users consulted on the study design.

Throughout the trial, participants in both treatment arms will receive standard asthma care in accordance with the national BTS/SIGN guidelines for the management of asthma in adults. No treatments or care will be withheld at any point in the study, with all participants (in either arm) able to receive any care considered appropriate by the treating physicians.

All participants completing at least 6 months of the trial follow-up period will be offered the option of having an active device in their home for a further 4 years, provided and maintained by the manufacturers Airsonett™.

### Funding source, sponsor and trial oversight

This study has been funded through a competitive grant application to the National Institute of Health Research (NIHR) Health Technology Assessment (HTA) funding stream. The sponsor for this trial is Portsmouth Hospitals NHS Trust. The device is supplied to the sponsor by the manufacturers Airsonett™. Under the terms of a comprehensive supply agreement with the sponsor, Airsonett™ will provide devices, installation and maintenance services and will install the devices directly into the participant’s homes. In collaboration with the sponsor, ORTU will oversee the quality assurance and trial conduct with routine and for-cause audit performed in accordance Good Clinical Practice guidelines as appropriate.

A Trial Management Group (TMG) including the Chief Investigator, Trial Coordinator, Trial Manager, Data Manager and Trial Statistician are in contact weekly.

A Trial Steering Committee (TSC) will meet at least 6 monthly, and more frequently if required, to review the trial progress and to ensure that it is being conducted in accordance with the protocol, relevant regulations and the principles of GCP.

A Data Safety and Monitoring Committee (DSMC) will review trial progress and safety data. The DSMC is independent of the trial investigators and will comprise three independent members including two clinical specialists and a trial statistician.

### Protocol amendments

Protocol amendments will be agreed upon with the Trial Steering Committee, Data Safety and Monitoring Committee, Sponsor and Funding Body prior to submission for ethical approval. Following ethical approval, protocol modifications will be communicated with relevant parties such as the trial investigators, the trial registry and, if required, trial participants.

### Dissemination policy

The results of the trial will be widely disseminated to patients, health professionals, commissioners, policy makers and the general public. Our patient public involvement members will play a key role in this. The trial results will be disseminated to a wide clinical audience through publication in the HTA Journal series and another high impact international peer‐reviewed scientific journal. The respiratory community will be targeted through presentations at international meetings of respiratory disease (British Thoracic Society, European Respiratory Society.) The commercial supplier, Airsonett, has also agreed to support a symposium at a scientific meeting to further disseminate the results.

## Discussion

The LASER Trial will address an important research question in severe allergic asthma.

To date, methods of allergen avoidance have lacked the necessary evidence to support their widespread implementation despite broncho-provocation experiments demonstrating that aeroallergens can induce bronchospasm, eosinophilic airway inflammation, and increased bronchial hyper-reactivity in sensitised patients.

A Cochrane review of house dust mite control measures in asthma concluded that chemical and physical methods aimed at reducing exposure to house dust mite allergens cannot be recommended. They recommended that if further studies were to be considered that they should be methodologically rigorous and use other methods than those used so far.

In designing the LASER Trial, we have attempted to address the question of whether this novel allergen intervention can reduce the frequency of severe exacerbations and improve asthma control and quality of life as compared to placebo, whilst being cost-effective and acceptable to adults with poorly controlled, severe allergic asthma. Using a rigorous methodological approach and with our primary outcome guided by our Patient Public Involvement members, we hope that we will be able to answer an important research question whilst keeping outcomes relevant to patients, clinicians, policy makers and commissioners.

In designing The LASER Trial, we have attempted to maintain a pragmatic approach to ensure as wide as possible external applicability of the results. We have had engagement from our PPI members at all stages of trial development from grant application through to trial design and delivery.

PPI members highlighted the importance of exacerbation frequency as our primary outcome measure as they are so important to patients with severe asthma, proving a constant threat to their ability to lead a normal life. PPI members guided the decision to collect diary data and peak flow recordings for 2 weeks prior to each follow up visit rather than throughout the 12-month treatment period in an attempt to reduce the burden on trial participants.

PPI members have also been integral in the design of the trial paperwork (participant information sheet, consent forms and diaries) and trial website. PPI review ensured that the information delivered was applicable to patients and that the plain English summaries were accessible to all.

PPI members were present at each of our patient information events, held to enhance recruitment to the trial. Patient representatives supported the trial team in delivering the presentation and fielding questions about the trial.

The eligibility criteria chosen for the trial were intended to be as inclusive as possible whilst reflecting normal clinical practice and ensuring internal validity and appropriate scientific rigor. The most current international respiratory society guidance was used for the definition of severe asthma and in defining severe exacerbations. Well-validated questionnaire tools and patient-reported outcome tools were chosen to monitor clinical outcomes.

### Trial status

The LASER Trial is now open to recruitment with 14 sites across England currently recruiting participants to the trial. Further information about the trial including updates on trial progress can be found at www.lasertrial.co.uk.

## Abbreviations

ACQ: Asthma Control Questionnaire
ACQoL: Adult Carer Quality of Life questionnaire
ANCOVA: Analysis of Covariance
AQLQ: Asthma Quality of Life Questionnaire
ATS: American Thoracic Society
BMI: Body Mass Index
COPD: Chronic Obstructive Pulmonary Disease
CPAP: Continuous Positive Airway Pressure
CRF: Case Report Form
DSMC: Data Safety and Monitoring Committee
EQ-5D-5L: EuroQol 5-Dimension 5-Level Questionnaire
ERS: European Respiratory Society
GCP: Good Clinical Practice
GETE: Global Evaluation of Treatment Effect
HTA: Health Technology Assessment
IgE: Immunoglobulin E
ITT: Intention To Treat
NHS: National Health Service
NIV: Non-Invasive Ventilation
ORTU: Oxford Respiratory Trials Unit
PEF: Peak Expiratory Flow
PI: Principal Investigator
PPI: Patient and Public Involvement
QALY: Quality Adjusted Life Year
SNOT-22: Sino-Nasal Outcome Test - 22 items
SPT: Skin Prick Testing
TLA: Temperature-controlled Laminar Airflow
TMG: Trial Management Group
TSC: Trial Steering Committee
WPAI-A: Work Productivity Activity Impairment questionnaire - Asthma
WPAI-CG: Work Productivity Activity Impairment questionnaire – Care Giver

## References

[1] Asthma UK. Living on a Knife Edge. A powerful and moving account of living with serious symptoms of asthma. London: Asthma UK, 2004.

[2] Holgate ST, Polosa R (2006). The mechanisms, diagnosis and management of severe asthma in adults. Lancet.

[3] Hoskins G, McCowan C, Neville RG, Thomas GE, Smith B, Silverman S (2000). Risk factors and costs associated with an asthma attack’. Thorax.

[4] Manson SC, Brown RE, Cerulli A, Vidaurre CF (2009). The cumulative burden of oral corticosteroid side effects and the economic implications of steroid use. Respir Med.

[5] Humbert M, Beasley R, Ayres J, Slavin R, Hébert J, Bousquet J (2005). Benefits of Omalizumab as add-on therapy in patients with severe persistent asthma who are inadequately controlled despite best available therapy (GINA 2002 step 4 treatment): INNOVATE. Allergy.

[6] National Institute for Health and Care Excellence. Omalizumab for treating severe persistent allergic asthma (review of technology appraisal guidance 133 and 201) [Internet] London, England: NICE; Apr, 2013. (NICE technology appraisal guidance 278). Available from: https://www.nice.org.uk/guidance/ta278.

[7] British Thoracic Society/Scottish Intercollegiate Guidelines Network. British guideline on the management of asthma. Thorax. 2014;69 (Suppl 1): 1-192. 25323740

[8] Bousquet J, Mantzouranis E, Cruz AA, Aït-Khaled N, Baena-Cagnani CE, Bleecker ER (2010). Uniform definition of asthma severity, control and exacerbations: document presented for the World Health Organisation consultation on Severe Asthma. J Allergy Clin Immunol.

[9] Heaney LG, Brightling CE, Menzies-Gow A, Stevenson M, Niven RM, British Thoracic Society Difficult Asthma Network (2010). Refractory asthma in the UK: cross-sectional findings from a UK multicentre registry. Thorax.

[10] Custovic A, Taggart SC, Francis HC, Chapman MD, Woodcock A (1996). Exposure to house dust mite allergens and the clinical activity of asthma. J Allergy Clin Immunol.

[11] Tunnicliffe WS, Fletcher TJ, Hammond K, Roberts K, Custovic A, Simpson A (1999). Sensitivity and exposure to indoor allergens in adults with differing asthma severity. Eur Respir J.

[12] Langley SJ, Goldthorpe S, Craven M, Morris J, Woodcock A, Custovic A (2003). Exposure and sensitisation to indoor allergens: Association with lung function, bronchial reactivity and exhaled nitric oxide measures in asthma. J Allergy Clin Immunol.

[13] Rosenstreich DL, Eggleston P, Kattan M, Baker D, Slavin RG, Gergen P (1997). The role of cockroach allergy and exposure to cockroach allergen in causing morbidity among inner-city children with asthma. NEJM.

[14] Custovic A, Simpson A, Chapman MD, Woodcock A (1998). Allergen avoidance in the treatment of asthma and atopic disorders. Thorax.

[15] Van Velzen E, van den Bos JW, Benckhuijsen JA, van Essel T, de Bruijn R, Aalbers R (1996). Effect of allergen avoidance at high altitude on direct and indirect bronchial hyperresponsiveness and markers of inflammation inchildren with allergic asthma. Thorax.

[16] Peroni DG, Boner AL, Vallone G, Antolini I, Warner JO (1994). Effective allergen avoidance at high altitude reduces allergen-induced hyperresponsiveness. Am J Respir Crit Care Med.

[17] Grootendorst DC, Dahlén SE, Van Den Bos JW, Duiverman EJ, Veselic-Charvat M, Vrijlandt EJ (2001). Benefits of high altitude allergen avoidance in atopic adolescents with moderate to severe asthma over and above treatment with high dose inhaled steroids. Clin Exp Allergy.

[18] Gøtzsche PC, Johansen HK. House dust mite control measures for asthma. Cochrane Database of Systematic Reviews. 2008, Issue 2. Art. No.: CD001187. DOI:10.1002/14651858.CD001187.pub3. 10.1002/14651858.CD001187.pub3 PMC8786269 18425868

[19] Sublett JL (2011). Effectiveness of air filters and air cleaners in allergic respiratory diseases: a review of the recent literature. Curr Allergy Asthma Rep.

[20] Sigsgaard T. Temperature regulated Laminair Airflow (TLA): TLA principles and practise, presented at European Academy of Allergy and Clinical Immunology 2010 Congress, London

[21] Gore RB, Boyle RJ, Hanna H, Custovic A, Gore C, Svensson P (2010). Personal allergen exposures are increased by changes in sleep position and improved by temperature controlled laminar airflow. Thorax.

[22] Sigsgaard T, Ravn P, Svensson P, Takai H. A comparison of the effectiveness of a temperature controlled laminar airflow device and a room air-cleaner in reducing particle concentrations in the breathing zone of a thermal manikin lying in a bed. 2010. Allergy. 65(Suppl. 92):694–756

[23] Boyle RJ, Pedroletti C, Wickman M, Bjermer L, Valovirta E, Dahl R (2012). Nocturnal Temperature Controlled Laminar Airflow for Treating Atopic Asthma: a randomised controlled trial. Thorax.

[24] Chung KF, Wenzel SE, Brozek JL, Bush A, Castro M, Sterk PJ (2013). International ERS/ATS guidelines on definition, evaluation and treatment of severe asthma. Eur Respir J.

[25] Reddel HK, Taylor DR, Bateman ED, Boulet LP, Boushey HA, Busse WW (2009). An Official American Thoracic Society/European Respiratory Society Statement: Asthma Control and Exacerbations. Standardizing Endpoints for Clinical Asthma Trials and Clinical Practice. Am J Respir Crit Care Med.

[26] Brooks R, Rabin R, Charro F. The measurement and valuation of health status using EQ-5D: a European perspective: evidence from the EuroQol BIO MED research programme. Rotterdam, The Netherlands: Kluwer Academic Publishers, Springer; 2003.

[27] Curtis L (2012). Unit costs of health and social care 2013.

[28] International Conference on Harmonisation of technical requirements for registration of pharmaceuticals for human use. ICH harmonised tripartite guideline. Guideline for Good Clinical Practice. Ottawa, Canada: Health Canada. 1996.

[29] Briggs A, Gray A (1998). The distribution of healthcare costs and their statistical analysis for economic evaluation. J Health Serv Res Policy.

[30] Claxton K, Sculpher M, McCabe C, Briggs A, Akehurst R (2005). Probabilistic sensitivity analysis for NICE technology assessment: not an optional extra. Health Econ.

[31] WMA Declaration of Helsinki - Ethical Principles for Medical Research Involving Human Subjects. 64th WMA General Assembly, Fortaleza, Brazil. World Medical Journal. 2013;59 (5):199.

